# On the distinction of empathic and vicarious emotions

**DOI:** 10.3389/fnhum.2013.00196

**Published:** 2013-05-15

**Authors:** Frieder M. Paulus, Laura Müller-Pinzler, Stefan Westermann, Sören Krach

**Affiliations:** ^1^Social Neuroscience Lab, Department of Psychiatry, Philipps-University MarburgMarburg, Germany; ^2^Department of Child and Adolescent Psychiatry, Philipps-University MarburgMarburg, Germany

**Keywords:** vicarious emotion, empathic emotion, anchoring, adjustment, vicarious embarrassment, mentalizing, mirroring, empathy

## Abstract

In the introduction to the special issue “The Neural Underpinnings of Vicarious Experience” the editors state that one “may feel embarrassed when witnessing another making a social faux pas”. In our commentary we address this statement and ask whether this example introduces a *vicarious* or an *empathic* form of embarrassment. We elaborate commonalities and differences between these two forms of emotional experiences and discuss their underlying mechanisms. We suggest that both, vicarious and empathic emotions, originate from the simulation processes mirroring and mentalizing that depend on anchoring and adjustment. We claim the term “empathic emotion” to be reserved exclusively for incidents where perceivers and social targets have shared affective experience, whereas “vicarious emotion” offers a wider scope and also includes non-shared affective experiences. Both are supposed to be highly functional in social interactions.

## Introduction

The human ability to infer others' emotions, thoughts or intentions is a central mechanism in creating meaningful social interactions. Accordingly, the question of how we develop a representation of our interaction partners' minds and emotions has been the focus of various disciplines such as social psychology, philosophy, anthropology, and biology. In the last decade the social neurosciences, specifically, have put tremendous efforts into disentangling the neural networks involved in this ability. Most of this research has concentrated on the phenomenon of “empathy.” Empathy has been defined as the state where people (i.e., *perceivers*^1^) represent the same emotion they are observing or imagining in another person (i.e., *social targets*) with full awareness that the source of their own experience is the other's emotion (De Vignemont and Singer, 2006). However, empathy only refers to a small amount of vicarious emotions people may experience while interacting with their social environment in everyday life (Singer and Lamm, 2009). With this commentary, we aim to broaden this perspective by proposing a clear-cut distinction between vicarious and empathic emotions, with the latter being a specific case of the first and both being mediated by two streams of simulation processes.

## Two processes of understanding others' emotions: mirroring and mentalizing

Mainly, two interacting processes have been proposed that allow perceivers to empathize (Keysers and Gazzola, 2007; Waytz and Mitchell, 2011). First, *mirroring* processes have been described as a direct mapping of another's observed actions and bodily states in one's own (i.e., the perceiver's) neural system that allow sharing the target's feelings in an embodied manner. Second, *mentalizing* processes which have been proposed to lead to comparable internal representations in perceivers, however, via a projection of oneself into the target's position (Keysers and Gazzola, 2007; Hein and Singer, 2008). Mentalizing thus involves imagining oneself in the same situation as the social target and helps to “intuitively” (Keysers and Gazzola, 2007) grasp the target's emotions as if they were one's own bodily states. These processes, mirroring and mentalizing, can be understood as forms of internal simulation that allow perceivers to experience another person's state on one's own body (see Waytz and Mitchell, 2011).

In order to shed light onto the neural mechanisms of these two processes to simulate the target's emotional state, the fundamental idea of these approaches is to compare neuronal networks involved in first-hand experiences of emotions or sensations (e.g., provoking pain or disgust through administration of electro-shocks or unpleasant odors, respectively) with the neuronal networks engaged while observing emotions or sensations in interaction partners (Wicker et al., 2003; Singer et al., 2004; Jabbi et al., 2007). Overlap in cortical activation between these experimental conditions is then interpreted as evidence for shared, “isomorphic”^2^ affective states between interaction partners and thus as a neuronal manifestation of empathy (Wicker et al., 2003; Gallese et al., 2004; Singer et al., 2004; Jackson et al., 2006). Irrespective of the underlying processes, neuroscience research has shown that the anterior insula and the anterior cingulate cortex are most robustly involved in common mapping of one's own and another's affective states during empathic experiences (Craig, 2009; Lamm and Singer, 2010).

Depending on the available input, perceivers rely on sensory [i.e., mirroring of gestures, mimics, bodily postures, sounds etc. in a near-simultaneous isomorphic fashion (Waytz and Mitchell, 2011)] and/or contextual information (i.e., mentalizing using semantic information, prior knowledge, past experiences in similar situations etc.) in order to represent another person's state (Waytz and Mitchell, 2011; Zaki and Ochsner, 2011). Among others, the premotor cortex and primary as well as higher order somatosensory cortices are thought to mediate the mirroring process (Avenanti et al., 2005). Mentalizing is typically associated with medial prefrontal cortex (mPFC), temporal pole, and superior temporal sulcus activation (Hein and Singer, 2008). Within the mentalizing network, the mPFC has been specifically linked to reflective processes about oneself and another (Mitchell et al., 2005) or imagining oneself in past and future events (Buckner and Carroll, 2007; Schacter et al., 2007). This supports the conceptualization of mentalizing as a process where perceivers project themselves into to the position of the social target.

## Dissociating vicarious and empathic emotions

The processes to infer the “physically invisible but psychologically real, internal state” (Zaki and Ochsner, 2011; p.159) can also result in “vicarious emotions” that are simulated in the absence of this specific emotional state in the social target. Although the terms “empathic emotions” (Batson, 1981; Lamm et al., 2007a; Hein and Singer, 2008; Pfeifer et al., 2008; Engen and Singer, 2012; Zaki and Ochsner, 2012) and “vicarious emotions” (Batson et al., 1987; Decety and Lamm, 2006; Keysers and Gazzola, 2009; Meyer et al., 2012; Niedenthal and Brauer, 2012) have been used with near identical meaning, we consider both concepts to have distinctive characteristics and consequences. This distinction is easily illustrated on the basis of vicarious embarrassment: in many social encounters perceivers feel vicariously embarrassed in the absence of embarrassment or any other emotion in the social target^3^ (Hawk et al., 2011; Krach et al., 2011; Müller-Pinzler et al., 2012; Paulus et al., 2013). Thus, the social target is unaware about the ongoing threats to her social integrity in this situation (Krach et al., 2011). Consequently, in contrast to empathic manifestations, vicarious embarrassment reflects an emotional state in the perceiver that does not match the internal, psychologically real state of the social target. Nonetheless, recent studies provided first evidence that similar processes of mentalizing and mirroring contribute to the perceiver's vicarious embarrassment (Hawk et al., 2011; Krach et al., 2011).

We have previously discussed how mentalizing can result in vicarious emotions that do not match the emotional state of the social target (Krach et al., 2011). This has been explained through self-projections of perceivers who transpose themselves into the position of others thereby integrating their own perspective within the mental simulation (Hawk et al., 2011). However, for several reasons, the mapping of the social target's state in the perceiver's neural network through mirroring processes is also not independent of the perceiver's perspective. First, similar to the processing of sensory information of one's own body (Gazzola et al., 2012), mirroring the target's state in a near-simultaneous isomorphic fashion is modulated by other processes such as mentalizing. This is particularly important in social contexts that constrain the desirability of displayed emotions (e.g., at work). In these situations the enacted and thus mirrored expressions could deviate from the corresponding internal psychological state. Second, the mirror neuron functioning is deeply integrated in a neural network that is tailored and tuned to process information of the perceiver's body. In the most extreme example this is illustrated with mirror neuron activity in response to observing robotic arms grasping objects (Gazzola et al., 2007; Keysers et al., 2010). Those robots do not have any human sensations or form intentions about their actions, however, the perceiver's neural system mirrors the action as if it was human. Consequently, depending on the idiosyncratic learning experiences the mirrored representation should deviate across different perceivers even if the inputs entering the system were exactly similar. These arguments illustrate how mirroring is indeed anchored in the characteristics of the perceiver's neural system and might be modulated by additional information accessible exclusively from the observer's perspective. The resulting simulation of the social target's state through mirroring processes could represent a genuine vicarious emotion. Previous research has already demonstrated such automated vicarious responses while e.g., observing numbed limbs that undergo biopsy (Lamm et al., 2007b).

These thoughts raise the question of whether vicarious in comparison to empathic emotional experiences may serve a useful function in social interactions or have to be considered as the result of immature and maladapted processes to representing another person's internal psychological state. With the help of some examples we argue that these vicarious emotions may indeed provide useful information for perceivers, enable helping behavior, and foster social interactions. First, vicarious emotions contribute to the social regulation of the perceiver's behavior. For instance, many forms of psychological punishment are used as an “example” to induce avoidance of disobedience from norms, even if the social target does not respond to the situation. Perceivers will nonetheless do so and vicariously experience the suffering in that situation. Second, imagine observing the above described non-embarrassed presenter who is currently unaware of the ongoing threat to her social integrity. For perceivers, their vicarious emotional response provides insights about the severity of the threat to the image of the social target. This internal vicarious representation of the unfavorable condition may help to motivate interventions from the perceivers' side in order to re-establish the social integrity of the target. In contrast, perceivers who are tied to an empathically accurate response that matches the internal psychological state of the target may be less prone to develop such motivations. Similarly, with regards to observing physical injuries to another's body, vicarious pain experiences, even in the absence of a psychological state of pain in the target, might provide vital information for initiating costly helping behaviors (Hein et al., 2010).

This line of argumentation supports the notion that human beings are not only passive perceivers in the context of social interactions but also active creators of shared emotional experiences. In a natural setting, they need to be aware of their own presence and the simulated vicarious emotions in response to another person's condition; is it in the presence or absence of an emotional state in the social target. The perceiver's construal of a social target's condition as the representation of an internal, “psychologically real” state might thus provide an unnecessarily narrow scope to examining vicarious emotions. Rather, vicarious emotions should be considered as the result of ongoing simulation processes that, depending on the social context as well as personal or task induced motivations are flexibly tuned to match another's internal psychological state.

**Figure 1:**
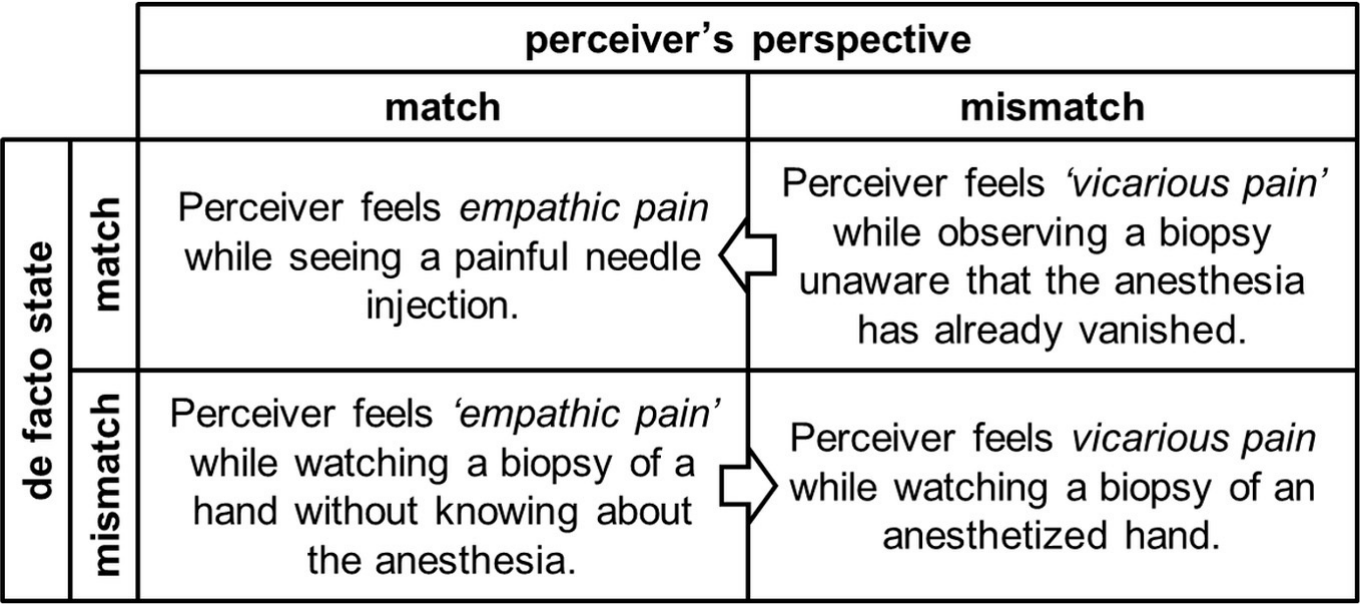
**Integrating the perceiver's perspective in vicarious and empathic emotions.** The figure illustrates how the perceiver's assumption about the match of her emotions with the social target's emotion may dissociate from the de facto state. Notably, the neural response pattern within each state is determined by the subjective appraisals of perceivers. The arrows indicate the adjustment of a subjectively “incorrect” stance during the course of social interactions (e.g., feedback of the social target) in order to match the demands of the social context. These transitions might specifically help to dissociate neural processes that are involved in the adjustment and anchoring of one's own perspective.

The question remains if perceivers, even if they intend to, always have correct assumptions about the emotion of the social target. Accordingly, social neuroscience has to consider the match or mismatch of the emotional experiences between social targets and perceivers from *two* perspectives: first, the de facto match or mismatch of the emotions between the perceiver and the social target, and second, the subjective stance of the perceiver about the match or mismatch with the social target's emotions. In social interactions both perspectives may occur independently of each other, resulting in four different states (for examples see Figure 1). The neural responses should not differ between de facto and subjective empathic and vicarious emotions, respectively. The transition from one of the states to another might nonetheless offer great potential for unraveling yet neglected neural processes in social interactions. This is especially important considering upcoming second-person neuroscience paradigms that allow the investigation of true social interactions (Krach et al., in press; Schilbach et al., in press).

## A process oriented perspective on vicarious emotions

**Figure 2:**
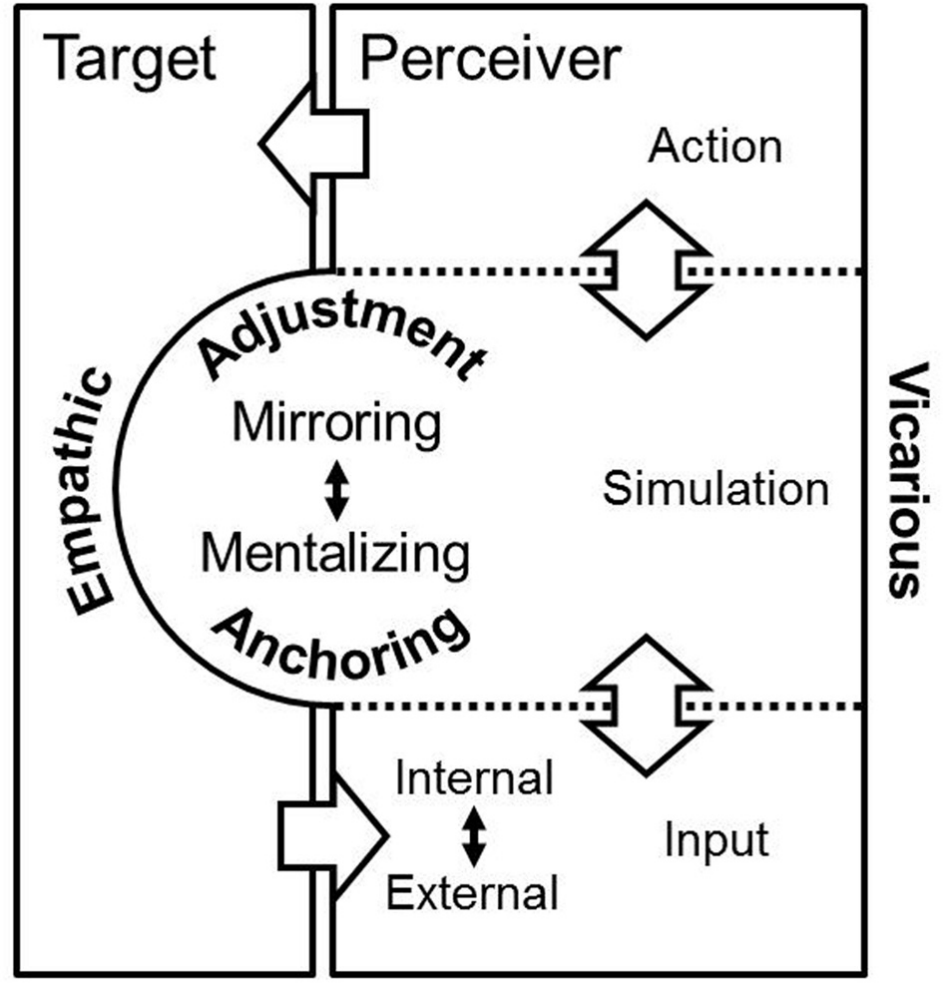
**Conceptualization of vicarious and empathic emotions in a unified framework.** The figure illustrates how perceiver and social target may interact in a shared social environment and how the perceiver represents vicarious and empathic emotions based on simulation processes. On the most abstract level, the input for the simulation stems from external (e.g., gesture, mimic, prosody) or internal sources (e.g., prior knowledge, past experiences with the interaction partner). Simulation of internal states is realized through two different streams, mirroring and mentalizing, which depend on the available input. Both streams of simulation are anchored in the perceiver's perspective and get adjusted to obtain the adequate outcome in the shared social environment. This can be rather empathic and/or vicarious emotional experience.

Ideas how to conceptually explain vicarious emotions can be derived from recent efforts in social psychology. Several behavioral studies have examined the process of understanding others' minds. Those studies indicate that people adopt others' perspectives by initially anchoring on their own perspective and then serially adjusting their internal representation to account for differences between themselves and others (Epley et al., 2004). This understanding has been mostly applied in context of cognitive inferences on another person's knowledge or attitudes but might be easily applicable for examining the neural underpinnings of vicarious emotions as well. In a shared social environment, perceivers have access to different inputs (i.e., internal and external, see Figure 2) allowing to simulate the social target's state. We have outlined above how both streams of simulation, mirroring and mentalizing, are anchored in the egocentric perspective of the perceiver. The social context then defines how the initial simulation needs to get adjusted in order to provide the foundation for successful social interactions. Depending on the appropriateness of the initial simulation (anchoring) the readjustment process might be more or less demanding and may finish after a “plausible” assessment is reached (Epley and Gilovich, 2001). Notably, the plausibility refers to both, vicarious and/or empathic emotional experiences (Figure 2).

So far, social neuroscience has predominantly investigated the two streams of simulation processes and their interactions (Zaki and Ochsner, 2012). We believe that focusing on the sub-processes of anchoring and adjusting in both streams of simulation has the potential to explain vicarious and empathic emotions in a parsimonious framework. A first fMRI study has indicated the potential for this approach in the social neurosciences (Tamir and Mitchell, 2010). While focusing on cognitive inferences, this study showed the mPFC to be specifically involved in the readjustment process during mentalizing. We would predict similar mPFC functioning in case of readjustment of vicarious emotions, both during mirroring or mentalizing processes. Extending on these findings, one can formulate more refined hypotheses on the involvement of neural networks in simulation processes and the specific functions of subunits within the system. These may allow differentiating vicarious and empathic emotions on the neural systems level and processes involved in the transitions from subjective to de facto vicarious or empathic states (see Figure 1). Here, we would predict the mPFC to play a pivotal role for remodeling the “incorrect” subjective state. Future studies on vicarious or empathic emotions, however, need to address the complexity of social situations and manipulate it to the extremes in order to elucidate the specific neural processes involved in the different stances.

Further, the modulatory role of contextual demands on brain and behavior can be tested. Among others, one could model the effects of time constraints or increased cognitive load on the perceiver side, or alter the perceiver's simulation by task induced manipulations. This understanding of simulation processes also is of clinical relevance. Instead of characterizing the impairments in both streams of simulation, research has to consider causes of clinical phenotypes on the level of anchoring and adjustment. The source of e.g., autistic symptomatology might rather originate from disturbed anchoring and adjustment and the inflexibility to modulate the simulation process according to social contextual demands (Paulus et al., 2013). Although, there is evidence for both simulation processes to be affected in individuals with autism (see Zaki and Ochsner, 2012) a theoretical work on autism-spectrum disorders also suggested that affected individuals have strong egocentrically anchoring that cannot be readjusted to the social target's perspective (De Vignemont and Frith, 2007) which might contribute to observed alterations in simulation processes.

In conclusion, we provide an argument for how to distinguish the terms “vicarious emotions” and “empathic emotions.” Both originate from the simulation processes mirroring and mentalizing, however, the term “empathic emotions” should be reserved only for incidents where perceivers and social targets have shared, “isomorphic” affective experience (Engen and Singer, 2012). Vicarious emotions offer a wider scope and also include non-shared affective experiences which are nonetheless highly functional in social interactions. With several examples we have briefly illustrated how the two streams of simulation, mirroring and mentalizing, are imbued by the perceiver's perspective which might result in both vicarious and/or empathic emotions. In order to explain these emotional experiences in a parsimonious framework, we think that anchoring and adjustment are the yet neglected concepts that need to get integrated into the research on the neural underpinnings of vicarious experience.

### Conflict of interest statement

The authors declare that the research was conducted in the absence of any commercial or financial relationships that could be construed as a potential conflict of interest.

## Footnotes

^1^Zaki and Ochsner (2011) described individuals focusing on someone else as “perceivers” and individuals being in the focus of the perceivers' attention as ‘social targets’. For the present article we take on this labeling and will refer to perceiver and social target in the following.

^2^In the neurosciences the term isomorphism might be understood with at least two different meanings: on the one hand, “isomorphic” patterns of information refer to the similar firing of mirror neurons during self-initiated actions and the observation of corresponding actions of others thus allowing computational predictions. On the other hand, in the context of empathy research the term “isomorphism” has been used to describe similar affective states between targets and perceivers (De Vignemont and Singer, 2006). Whereas the earlier usage refers to the micro-level of information processing in the brain, the latter describes the subjective level of affective experiences. In the present manuscript we use the term “isomorphism” with the latter meaning.

^3^For example, vicarious embarrassment is experienced by attendees of a scientific conference when they observe the presenter of a talk returning from the rest room not realizing that toilet paper is sticking out of the back of her pants.
